# Association Between Intensive Care Unit-Acquired Weakness and Early Nutrition and Rehabilitation Intensity in Mechanically Ventilated Patients: A Multicenter Retrospective Observational Study

**DOI:** 10.7759/cureus.37417

**Published:** 2023-04-11

**Authors:** Shinichi Watanabe, Jun Hirasawa, Yuji Naito, Motoki Mizutani, Akihiro Uemura, Shogo Nishimura, Keisuke Suzuki, Yasunari Morita, Yuki Iida

**Affiliations:** 1 Department of Physical Therapy, Gifu University of Health Science, Gifu, JPN; 2 Department of Rehabilitation Medicine, Tosei General Hospital, Seto, JPN; 3 Department of Rehabilitation Medicine, Shizuoka Medical Center, Shizuoka, JPN; 4 Department of Rehabilitation Medicine, Ichinomiya Nishi Hospital, Ichinomiya, JPN; 5 Department of Rehabilitation Medicine, Toyohashi Municipal Hospital, Toyohashi, JPN; 6 Department of Rehabilitation Medicine, Kainan Hospital, Yatomi, JPN; 7 Department of Emergency Medicine, Nagoya Medical Center, Nagoya, JPN; 8 Department of Physical Therapy, Toyohashi Sozo University, Toyohashi, JPN

**Keywords:** critical thinking, intensive care unit stay, early rehabilitation, enteral feeding, calorie intake, early mobilization, icu-acquired weakness, physical medicine and rehabilitation, mechanical ventilation, early nutrition

## Abstract

Background: Muscle weakness in the intensive care unit (ICU), referred to as ICU-acquired weakness (ICUAW), is a common complication observed in patients receiving mechanical ventilation. This study aimed to investigate whether rehabilitation intensity and nutrition during ICU admission are associated with the incidence of ICUAW.

Materials and methods: Consecutive patients aged ≥18 years who were admitted to the ICU between April 2019 and March 2020 and who received mechanical ventilation for >48 h were eligible. The included patients were divided into two groups: the ICUAW group and the non-ICUAW group. ICUAW was designated by a Medical Research Council score of less than 48 during discharge from the ICU. Patient characteristics, time to achieve ICU mobility scale (IMS) 1 and IMS 3, calorie and protein deliveries, and blood creatinine and creatine kinase levels were evaluated as study data. In this study, the target dose for the first week after admission to the ICU at each hospital was set at 60-70% of the energy requirement calculated by the Harris-Benedict formula. Univariate and multivariate analyses were used to determine the odds ratios (OR) for each factor and to explain the risk factors for the occurrence of ICUAW at ICU discharge.

Results: During the study period, 206 patients were enrolled; 62 of the 143 included patients (43%) had ICUAW. The results of multivariate regression analysis showed that low time to IMS 3 achievement (OR 1.19, 95% confidence interval (CI) 1.01-1.42, p=0.033), and high mean calorie (OR 0.83, 95% CI 0.75-0.93, p<0.001) and protein deliveries (OR 0.27, 95% CI 0.13-0.56, p<0.001) were independently associated with the occurrence of ICUAW.

Conclusions: Increase in rehabilitation intensity and mean calorie and protein deliveries were associated with a decrease in the occurrence of ICUAW at ICU discharge. Further research is required to validate our results. Our observations, increasing the intensity of physical rehabilitation and the average calorie and protein delivery levels during ICU stay, appear to be the preferred strategies for achieving non-ICUAW.

## Introduction

In recent years, the survival rate of patients admitted to the intensive care unit (ICU) has improved with progress in medical treatment [1]. Patients with residual motor dysfunction in the acute phase of a serious illness and mechanically ventilated patients during the course of treatment often require long-term rehabilitation even after discharge from the ICU [2,3]. Dysfunction in the ICU, especially muscle weakness, is called ICU-acquired weakness (ICUAW). ICUAW causes muscle weakness within a few days of the onset of a severe illness and develops in a different course than disuse syndrome [4,5]. ICUAW is strongly associated with severe acute illness, causing dysfunction and increased mortality [5]. Even in patients who have survived a serious illness, dysfunction can continue for a long time after ICU discharge, and can severely affect their quality of life; its prevention is, therefore, essential [6,7].

The major risk factors for ICUAW have been reported to be the severity and duration of systemic inflammatory reactions, use of mechanical ventilation, drug effects, inactivities such as bed rest, and malnutrition [8-10]. Of these, the risk factors that can be improved relatively easily are rest and undernutrition in the forced supine position [11]. The evidence for early mobilization preventing ICUAW is limited, but previous studies have shown that early mobilization improves physical function after discharge and helps patients recover [12,13].

Mobilization and nutritional therapy are important for minimizing ICUAW. According to previous studies, patients' outcomes are better when they reach their targeted caloric intake during their stay in the ICU [14]. The administration of protein is also necessary to maintain muscle mass and is regarded as a key target in this regard [15]. Therefore, we believe that it is desirable to start nutritional therapy and carry out early mobilization of critically ill patients after appropriate evaluation of hemodynamics and intestinal function is performed immediately after admission to the ICU. These adjustments may also be important for ICUAW prevention. However, the association between nutrition and mobilization levels for the development of ICUAW has not yet been fully investigated [16].

Providing evidence of the appropriate timing of intensity rehabilitation initiation and nutritional intake for early recovery may help prevent ICUAW in critically ill patients. Therefore, we hypothesized that the intensity of early mobilization and nutritional intake during ICU stay in mechanically ventilated patients is significantly associated with the incidence of ICUAW. This study aimed to investigate whether rehabilitation intensity and nutrition during ICU admission are associated with the incidence of ICUAW.

## Materials and methods

Study design and setting

This study was a secondary analysis of a dataset acquired in a previous study investigating the association between mobilization barriers and activity in independent daily living at discharge during the first week of ICU stay [17]. All patients who were admitted into the ICUs of any one of the six tertiary hospitals in Japan between April 2019 and March 2020 were screened. The exclusion criteria were as follows: patients aged <18 years, unable to walk independently before hospitalization, neurologically impaired, incapable of communicating, with a terminal/end-of-life status, or had data loss. All other patients were included.

The included patients were divided into two groups: the ICUAW group and the non-ICUAW group. ICUAW was designated by a Medical Research Council score (assessed by a physiotherapist) less than 48 during discharge from the ICU [18].

Nutrition therapy

Enteral nutrition was initiated via the nasogastric route within 48 hours of admission, provided that there were no contraindications, with a rate of 10 to 20 mL/h, and was incrementally escalated. Detailed intake criteria were applied at the discretion of the attending medical practitioner, contingent upon the patient's clinical status and the severity of their ailment, and the circumstances of the participating facilities. The actual weight was used for calculating nutritional values. A hospital meal was provided when the patient recovered and could eat orally. All participating hospitals are following the 2016 Japanese version of nutrition guidelines for critically ill patients [19]. In this study, the target dose for the first week after admission to the ICU at each hospital was set at 60-70% of the energy requirement calculated by the Harris-Benedict formula.

Rehabilitation

The initial morning after admission to the ICU was identified as the first day, and rehabilitation following ICU admission was carried out based on a predetermined protocol. The first morning after admission to the ICU was defined as the first day, and rehabilitation after ICU admission was performed according to a protocol. The physiotherapist intervened only on weekdays, and the nurse rehabilitated the patient in bed on weekends. The protocol established five levels of rehabilitation (level 1: passive exercise and respiration physiotherapy; level 2: active exercise; level 3: sitting; level 4: standing; and level 5: walking) based on the patient's medical condition [7,20-22]. At each participating hospital, ICU practitioners or physiotherapists made the final determination concerning the degree of rehabilitation based on the patient's status and by referring to the protocol. This protocol has been used in routine practice in multiple centers, and validation of the protocol’s safety has already been reported [23]. In this study, we sought to mobilize all patients equally and daily under the protocol tailored to each participating hospital. All patients were expected to partake in at least one 20-minute rehabilitation session each day on weekdays. All patients underwent rehabilitation on weekdays by a physiotherapist or occupational therapist, focusing on exercises that targeted muscle strengthening, balance, and ambulation, including climbing stairs. This approach followed the rehabilitation policy of the general ward of each hospital and was implemented subsequent to the patient's discharge from the ICU.

Data collection

The physiotherapist daily documented the rehabilitation session data, comprising the highest score on the ICU mobility scale (IMS), time taken to achieve IMS 1 and IMS 3, and the mean IMS score throughout the ICU stay. The IMS is an 11-point ordinal scale with scores ranging from 0 (absence of mobilization) to 10 (independent ambulation), which is a sensitive tool for measuring mobility outcomes in critically ill patients [24]. The nutritional outcomes were evaluated by assessing calorie and protein intake. The hospital nutritionist calculated the total calorie and protein deliveries on a daily basis, while nutritional intake in patients consuming oral food was assessed using the hospital-ready reckoners and the recorded food intake. We recorded the blood creatinine level as an indicator of renal function and creatine kinase level as an indicator of energy metabolism. The baseline characteristics of the patients were recorded, encompassing age, sex, body mass index (BMI), Charlson comorbidity index, pre-hospitalization Barthel index, admission diagnosis to ICU, Acute Physiology and Chronic Health Evaluation II (APACHE II) score, Sequential Organ Failure Assessment (SOFA) score, rate of enteral nutrition, length of stay at the ICU, the duration of mechanical ventilation, vasopressor utilization, neuromuscular blocking agent administration, dialysis, and laboratory data on day 1.

Statistical analysis

Continuous variables were presented as medians (interquartile range (IQR)) due to their non-normal distribution, whereas categorical variables were displayed as numbers and percentages. The chi-square test was utilized for categorical covariates and the Mann-Whitney U-test was employed for comparing two groups (ICUAW and non-ICUAW groups).

Univariate and multivariate analyses were conducted to ascertain the odds ratios (OR) for each factor and to explicate the risk factors of ICUAW at ICU discharge. To determine the influence of the relationship between the outcomes, variables with p-values <0.10 in the univariate analysis were entered into a multivariate analysis. In order to prevent collinearity, the correlation coefficients between each parameter were calculated and verified to demonstrate a lack of high correlation. To evaluate mobilization, a Kaplan-Meier curve was generated to display the proportion of patients who attained IMS 1 and IMS 3 by the time of ICU discharge, and differences were examined utilizing a log-rank test.

All analyses were conducted utilizing JMP (version 13.0; SAS Institute Inc., Cary, NC, USA). Statistical tests were two-tailed, and statistical significance was established at p <0.05.

Ethics approval and consent to participate

Ethics approval and consent for participation were obtained for this multicenter retrospective cohort study from the Ethics Committee of Nagoya Medical Center (approval number: 2021-012). The study was performed in accordance with the principles outlined in the Declaration of Helsinki, and the necessity for informed consent in accordance with national regulations was waived by the institutional review board mentioned above, given that this was a retrospective cohort study. The identities of human participants and other identifying information were not employed throughout the study process and were not featured in any portion of the manuscript.

## Results

Baseline patient characteristics and course factors

**Figure 1:**
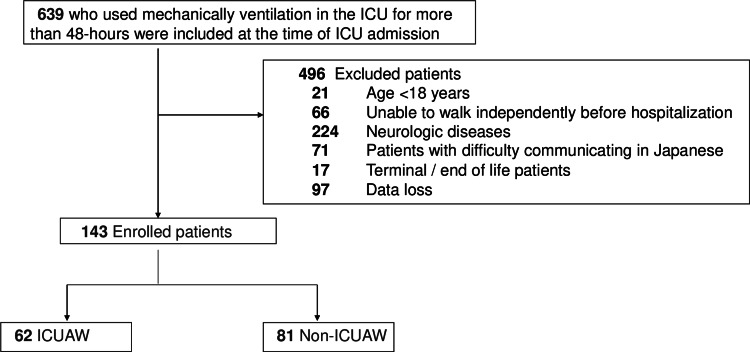
Flow chart ICU: intensive care unit; ICUAW: ICU-acquired weakness

Out of the 639 individuals who were initially enlisted in the research, 496 of them were eliminated. The exclusions comprise 21 patients who were aged < 18 years, 66 patients who were unable to walk independently before hospitalization, 224 patients who had neurological diseases, 71 patients with difficulty communicating in Japanese, 17 patients with terminal/end-of-life conditions, and 97 patients whose data were lost (Figure 1).

The baseline characteristics and course factors of the study population during hospitalization are shown in Table 1 and Table 2. Sixty-two of the 143 patients (43%) had ICUAW. The ICUAW group had a significantly lower BMI than the non-ICUAW group, a lower Barthel index before hospitalization score, a higher APACHE score, and higher creatinine levels at ICU admission.

**Table 1:** Comparison of patient characteristics based on intensive care unit-acquired weakness. Data are presented as median (interquartile range) or number (%). ICUAW: intensive care unit-acquired weakness; IQR: interquartile range; BMI: body mass index; CCI: Charlson comorbidity index; BI: Barthel index; APACHE: Acute Physiology and Chronic Health Evaluation; SOFA: Sequential Organ Failure Assessment

Variables	All Patients n=143	ICUAW n=62	Non-ICUAW n=81	P value
Age (years), median (IQR)	70.0 (59.0-77.0)	69.0 (56.8-76.0)	70.0 (59.5-78.0)	0.595
Gender (male), n (%)	97 (68)	38 (61)	59 (73)	0.262
BMI (kg/m^2^), median (IQR)	22.9 (19.5-26.3)	21.3 (18.3-25.3)	23.6 (20.3-26.9)	0.021
CCI, median (IQR)	1.0 (0-3.0)	1.0 (0-2.0)	1.0 (0-3.0)	0.875
BI before hospitalization, n (%)	100 (95-100)	100 (90-100)	100 (100-100)	0.029
ICU admission diagnosis, n (%)				
Acute respiratory failure	25 (17)	9 (14)	16 (20)	0.259
Cardiovascular disease	46 (32)	16 (26)	30 (37)	
Gastric or colonic surgery	32 (22)	16 (26)	16 (20)	
Sepsis, non-pulmonary	12 (9)	8 (13)	4 (5)	
Other diagnoses	28 (20)	13 (21)	15 (19)	
APACHE II score, median (IQR)	24.0 (18.8-29.0)	24.5 (20.0-31.0)	23.5 (17.0-27.5)	0.033
SOFA at ICU admission, median (IQR)	8.0 (7.0-10.0)	8.5 (7.0-12.0)	8.0 (7.0-10.0)	0.114
Laboratory data on day 1				
Creatinine, mg/dL, median (IQR)	1.2 (0.9-1.7)	1.6 (1.0-2.4)	1.1 (0.8-1.5)	0.002
Creatine Kinase, IU/L, median (IQR)	272 (108-802)	251 (114-1355)	276 (98-654)	0.310

**Table 2:** Comparison of course factors during hospitalization based on the occurrence of intensive care unit-acquired weakness. Data are presented as median (interquartile range) or number (%). IQR: interquartile range; BMI: body mass index; CCI: Charlson comorbidity index; BI: Barthel index; ICU: intensive care unit; APACHE: Acute Physiology and Chronic Health Evaluation; SOFA: Sequential Organ Failure Assessment; IMS: ICU mobility scale

Variables	All Patients n=143	ICUAW n=62	Non-ICUAW n=81	P value
Mobilization level				
IMS 1 achieved, day, median (IQR)	2.0 (1.0-3.0)	2.0 (1.0-3.0)	1.0 (1.0-3.0)	0.155
IMS 3 achieved, day, median (IQR)	5.0 (3.0-8.0)	7.0 (4.0-10.0)	4.0 (3.0-6.0)	<0.001
Highest IMS during ICU stay, median (IQR)	5.0 (3.0-7.0)	3.0 (1.0-5.0)	6.0 (3.0-8.0)	<0.001
Mean IMS during ICU stay, median (IQR)	3.0 (2.2-5.5)	1.5 (0.6-2.8)	5.0 (3.0-7.0)	<0.001
Nutrition				
Mean calorie delivery, kcal/kg/day, median (IQR)	5.7 (1.2-9.5)	2.6 (0-7.0)	8.2 (4.8-12.1)	<0.001
Mean protein delivery, g/kg/day, median (IQR)	0.6 (0.2-1.1)	0.4 (0-0.7)	0.8 (0.5-1.4)	<0.001
Enteral nutrition, n (%)	83 (58)	39 (63)	44 (54)	0.312
Parental nutrition, n (%)	70 (49)	28 (45)	42 (52)	0.427
Treatment				
ICU length of stay, median (IQR)	6.9 (5.0-9.4)	7.5 (5.2-11.6)	6.5 (4.8-8.9)	0.059
Duration of MV, d, median (IQR)	4.2 (2.9-6.8)	5.3 (3.2-9.1)	3.7 (2.8-5.1)	0.001
Steroid use, n (%)	48 (34)	23 (37)	25 (31)	0.434
Vasopressor use, n (%)	127 (89)	54 (87)	73 (90)	0.601
Neuromuscular blocking agent use, n (%)	26 (18)	15 (25)	11 (14)	0.127
Dialysis use, n (%)	40 (28)	25 (40)	15 (19)	0.005
Laboratory data				
Mean creatinine, mg/dL, median (IQR)	1.1 (0.7-1.7)	1.3 (0.8-2.5)	0.9 (0.7-1.3)	0.005
Mean Creatine Kinase, IU/L, median (IQR)	246 (122-585)	344 (157-960)	222 (98-433)	0.014

In the comparison of course factors during hospitalization, the ICUAW group had a significantly shorter time to achieve IMS 3 than the non-ICUAW group, lower highest and mean IMS achieved during ICU stay, longer duration of mechanical ventilation, higher rate of dialysis use, and lower mean creatinine and creatine kinase levels.

Association between ICUAW and patient characteristics and course factors

After performing the univariate analysis, BMI, APACHE II score, time to achieve IMS 3, highest IMS, mean IMS, mean calorie delivery, mean protein delivery, duration of mechanical ventilation, and dialysis use were included in the multivariate regression analysis (Table 3). The results showed that low APACHE II (OR 1.10, 95% confidence interval (CI) 1.03-1.18, p = 0.002), low time to achieve IMS 3 (OR 1.19, 95% CI 1.01-1.42, p = 0.033), high mean calorie delivery (OR 0.83, 95% CI 0.75-0.93, p < 0.001), and high protein delivery (OR 0.27, 95% CI 0.13-0.56, p < 0.001) were independently associated with the occurrence of ICUAW.

**Table 3:** Predictors of occurrence of the intensive care unit-acquired weakness, according to the univariate and multivariate regression analyses. CI: confidence intervals; ICU: intensive care unit; IMS: ICU mobility scale; BMI: body mass index; CCI: Charlson comorbidity index; BI: Barthel index; APACHE: Acute Physiology and Chronic Health Evaluation; SOFA: Sequential Organ Failure Assessment; MV: mechanical ventilation

Variables	Univariable analysis			Multivariable analysis		
	Odds ratio	95% CI	p value	Odds ratio	95% CI	p value
Age (every 1-year increase)	0.99	0.98-1.02	0.965			
male	0.59	0.29-0.96	0.145			
BMI (every 1-kg/m^2^ increase)	0.93	0.86-0.99	0.046	0.97	0.88-1.08	0.652
CCI (every 1 increase)	1.01	0.83-1.23	0.883			
BI before hospitalization (every 1 increase)	0.98	0.95-1.02	0.357			
Acute respiratory failure	0.69	0.28-1.69	0.416			
Cardiovascular disease	0.59	0.29-1.22	0.156			
Gastric or colonic surgery	1.41	0.64-3.11	0.391			
Sepsis, non-pulmonary	2.85	0.82-9.95	0.100			
APACHE II score (every 1 increase)	1.06	1.01-1.11	0.017	1.10	1.03-1.18	0.002
SOFA at ICU admission (every 1 increase)	1.09	0.98-1.22	0.100			
Creatinine on day 1 (every 1-mg/dL increase)	1.28	0.97-1.67	0.055			
Creatine Kinase on day 1 (every 1-IU/L increase)	1.01	0.99-1.01	0.071			
IMS 1 achieved (every 1-day increase)	1.21	0.98-1.50	0.073			
IMS 3 achieved (every 1-day increase)	1.27	1.13-1.43	<0.001	1.19	1.01-1.42	0.033
Highest IMS during ICU stay (every 1 increase)	0.72	0.61-0.87	<0.001	0.93	0.56-1.38	0.785
Mean IMS during ICU stay (every 1 increase)	0.54	0.41-0.72	<0.001	0.92	0.58-1.44	0.716
Mean calorie delivery (every 1- kcal/kg/day increase)	0.85	0.78-0.91	<0.001	0.83	0.75-0.93	<0.001
Mean protein delivery (every 1-g/kg/day increase)	0.35	0.19-0.64	<0.001	0.27	0.13-0.56	<0.001
Enteral nutrition	1.43	0.73-2.82	0.302			
Parental nutrition	0.76	0.39-1.48	0.427			
ICU length of stay (every 1-day increase)	1.08	0.97-1.20	0.145			
Duration of MV (every 1-day increase)	1.08	1.00-1.16	0.027	0.99	0.90-1.09	0.823
Steroid use	1.32	0.66-2.66	0.435			
Vasopressor us	0.74	0.26-2.10	0.570			
Neuromuscular blocking agent us	2.03	0.86-4.91	0.104			
Dialysis use	2.98	1.40-6.33	0.047	1.33	0.49-3.56	0.581
Mean creatinine, mg/dL (every 1-mg/dL increase)	1.28	0.91-1.89	0.204			
Mean Creatine Kinase (every 1-IU/L increase)	1.01	0.99-1.01	0.347			

Comparison of therapeutic outcomes in rehabilitation and nutrition

**Figure 2:**
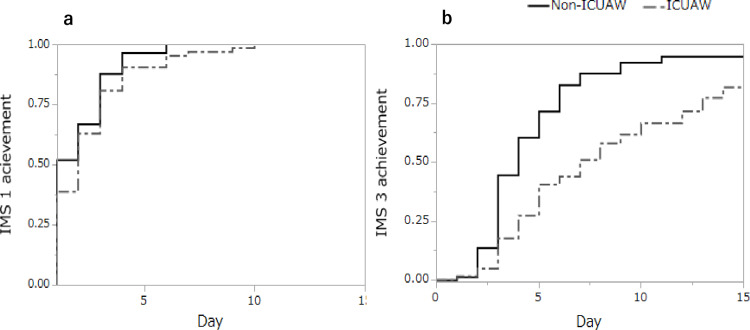
Comparison of therapeutic outcomes in rehabilitation. a Kaplan-Mayer curves for IMS 1, b Kaplan-Mayer Curves for IMS 3. The Kaplan-Meier curve showed that the non-ICUAW group achieved IMS 3 earlier (p<0.001), whereas no significant difference was observed in the achievement of IMS 1 (p=0.106). IMS: ICU mobility scale; ICUAW: intensive care unit-acquired weakness

The Kaplan-Meier curve showed that the non-ICUAW group achieved IMS 3 earlier (p<0.001), whereas no significant difference was observed in the achievement of IMS 1 (p=0.106) (Figure 2).

**Figure 3:**
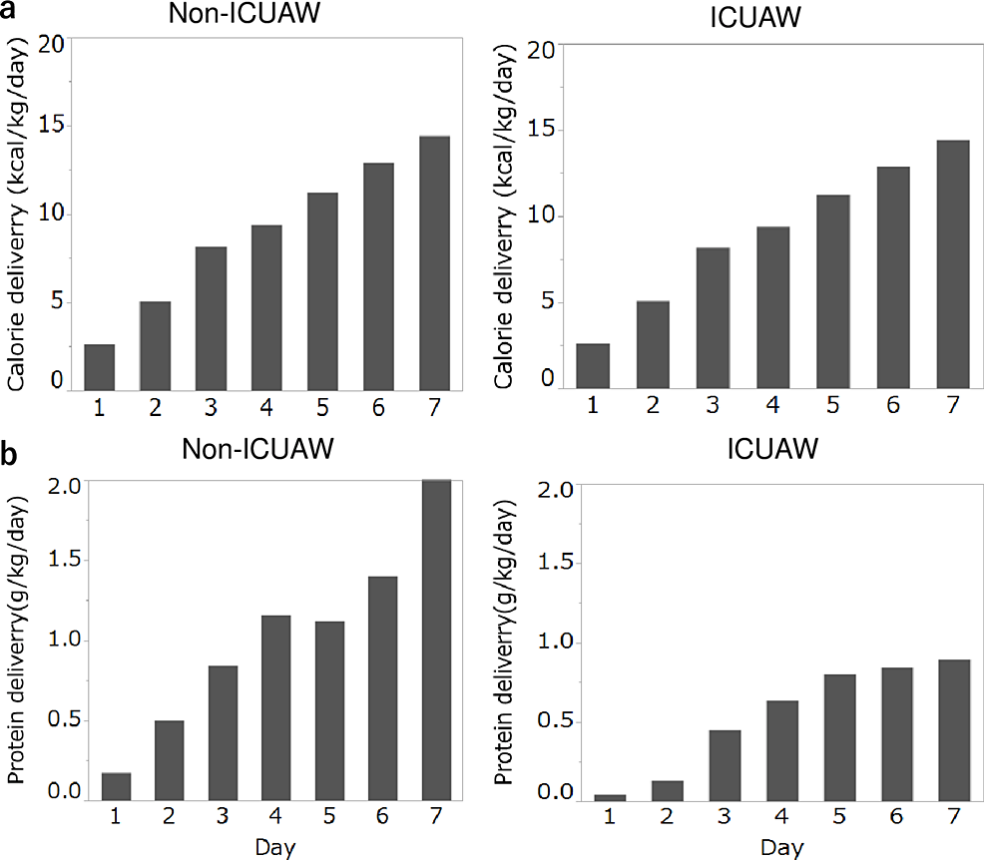
Comparison of therapeutic outcomes in nutrition. a daily trajectory of mean calorie delivery, and b daily trajectory of mean protein delivery. Mean calorie delivery (2.6 vs. 8.2 kcal/kg/day, p<0.001) and mean protein delivery (0.4 vs. 0.8 g/kg/day, p<0.001) from ICU stay were higher in the non-ICUAW group. ICUAW: intensive care unit-acquired weakness

Mean calorie delivery (2.6 vs. 8.2 kcal/kg/day, p<0.001) and mean protein delivery (0.4 vs. 0.8 g/kg/day, p<0.001) from ICU stay were higher in the non-ICUAW group (Figure 3).

## Discussion

As the survival rates of serious illnesses continue to improve, an increasing emphasis is being placed on improving the outcomes of these survivors. ICUAW is known to adversely affect both short-term and long-term outcomes; it is, therefore, important to understand its etiology and identify patients at the greatest risk of developing neuromuscular weakness [9,25]. This retrospective cohort study was conducted across multiple centers in Japan, utilizing patient data from the ICUs of six tertiary hospitals. Our study aimed to investigate the incidence of ICUAW of mechanically ventilated patients within the ICUs. Additionally, we examined the association between the time required to achieve IMS 3, average caloric intake, and protein intake. In a population of severely mechanically ventilated patients, the time to achieving IMS 3 has been shown to be independently associated with the occurrence of ICUAW at ICU discharge. In fact, early achievement of higher-intensity rehabilitation or sitting on the edge of the bed significantly reduced the likelihood of developing neuromuscular weakness.

ICUAW affects not only mortality but also health-related quality of life, extending the length of ICU stay [7,9,25]. Deep sedation has been associated with ICUAW. In addition, there is considerable evidence that early rehabilitation strategies with minimal sedation can reduce the incidence of ICUAW [12,13,26], consistent with previous studies. However, no significant association was observed with the occurrence of ICUAW based on the maximum or average intensity of rehabilitation. This may be due to the different evaluation times for each patient, as the ICUAW diagnosis was set at the time of ICU discharge. The analysis of physical function, encompassing both post-discharge and post-discharge recovery, over an extended period of time may result in more precise evaluations. Furthermore, patient severity variances may have impacted the outcomes.

In this study, the non-ICUAW group was able to significantly increase the delivery of inoculated calories and proteins, even after controlling for undereating, compared to the ICUAW group. Despite not meeting the overall target of 1.8 g/kg/day for protein intake, the average protein delivery increased to over 1.0 g/kg/day after four days. The gradual augmentation of protein delivery after day 3 is believed to enhance patient prognosis [27], as indicated in the nutritional guidelines that advocate for a target of at least 1.3 g/kg/day following gradual increments [28].

Previous studies have indicated that administering high levels of protein in the acute phase can have detrimental effects on muscles and that pre-day 3 administration may worsen the patient's prognosis [26]. In addition, avoiding parenteral nutrition in the first week of ICU admission reduced weakness [29]. This relationship could not be confirmed because swallowing was not systematically evaluated. In the current study, early protein overdose may have had adverse effects without a significant improvement in physical function. Optimal protein delivery is controversial, but future research should require protocols to set calorie and protein targets and validate their effectiveness. The present study could not evaluate the nitrogen balance. Nitrogen balance is independently associated with muscle loss and may serve as a guide for protein delivery [16].

Several limitations were observed in this study that warrant attention. The primary limitations were the small sample size and comparability of the two groups, which may restrict the generalizability of our findings to other ICUs. The presence of unadjusted confounding factors, such as medication and ventilator settings, further adds to the limitations. Different pressure and volume of ventilatory support required different doses of nutrition, which may have influenced the results of this study. Additionally, the decision of whether a patient could receive rehabilitation at a higher level depended on the rehabilitation policy of each participating hospital. This study was unable to examine the dosage and type of exercises performed. Therefore, it was challenging to identify the reason behind the unavailability of early mobilization, whether it was due to poor general conditions or other factors. Furthermore, in this study, we were unable to investigate the frequency of use of neuromuscular electrical stimulation and cycle ergometry, which may have influenced the incidence of ICUAW. A multicenter, prospective cohort study that includes all mechanically ventilated patients is likely to resolve the remaining queries. Thus, the importance of this protocol needs further examination in future studies.

## Conclusions

An increase in rehabilitation intensity and the mean calorie and protein deliveries were associated with a decrease in the occurrence of ICUAW at ICU discharge. Our observations were that accelerating the start of physical rehabilitation that requires more strength than that required to sit on the edge of the bed from admission to the ICU and increasing the average calorie and protein delivery levels appear to be the preferred strategies for preventing ICUAW. Further research is needed to identify and eliminate the confounding factors involved in early mobilization and nutritional therapy.
